# Most Popular Signal Processing Methods in Motor-Imagery BCI: A Review and Meta-Analysis

**DOI:** 10.3389/fninf.2018.00078

**Published:** 2018-11-06

**Authors:** Piotr Wierzgała, Dariusz Zapała, Grzegorz M. Wojcik, Jolanta Masiak

**Affiliations:** ^1^Department of Neuroinformatics, Faculty of Mathematics, Physics and Computer Science, Institute of Computer Science Maria Curie-Sklodowska University, Lublin, Poland; ^2^Department of Experimental Psychology The John Paul II Catholic University of Lublin, Lublin, Poland; ^3^Neurophysiological Independent Unit of the Department of Psychiatry Medical University of Lublin, Lublin, Poland

**Keywords:** brain-computer interfaces, motor imagery, electroencephalography, meta-analysis, sensorimotor rhythms

## Abstract

Brain-Computer Interfaces (BCI) constitute an alternative channel of communication between humans and environment. There are a number of different technologies which enable the recording of brain activity. One of these is electroencephalography (EEG). The most common EEG methods include interfaces whose operation is based on changes in the activity of Sensorimotor Rhythms (SMR) during imagery movement, so-called Motor Imagery BCI (MIBCI).The present article is a review of 131 articles published from 1997 to 2017 discussing various procedures of data processing in MIBCI. The experiments described in these publications have been compared in terms of the methods used for data registration and analysis. Some of the studies (76 reports) were subjected to meta-analysis which showed corrected average classification accuracy achieved in these studies at the level of 51.96%, a high degree of heterogeneity of results (*Q* = 1806577.61; *df* = 486; *p* < 0.001; *I*^2^ = 99.97%), as well as significant effects of number of channels, number of mental images, and method of spatial filtering. On the other hand the meta-regression failed to provide evidence that there was an increase in the effectiveness of the solutions proposed in the articles published in recent years. The authors have proposed a newly developed standard for presenting results acquired during MIBCI experiments, which is designed to facilitate communication and comparison of essential information regarding the effects observed. Also, based on the findings of descriptive analysis and meta-analysis, the authors formulated recommendations regarding practices applied in research on signal processing in MIBCIs.

## 1. Introduction

### 1.1. Rationale

**Figure 1:**
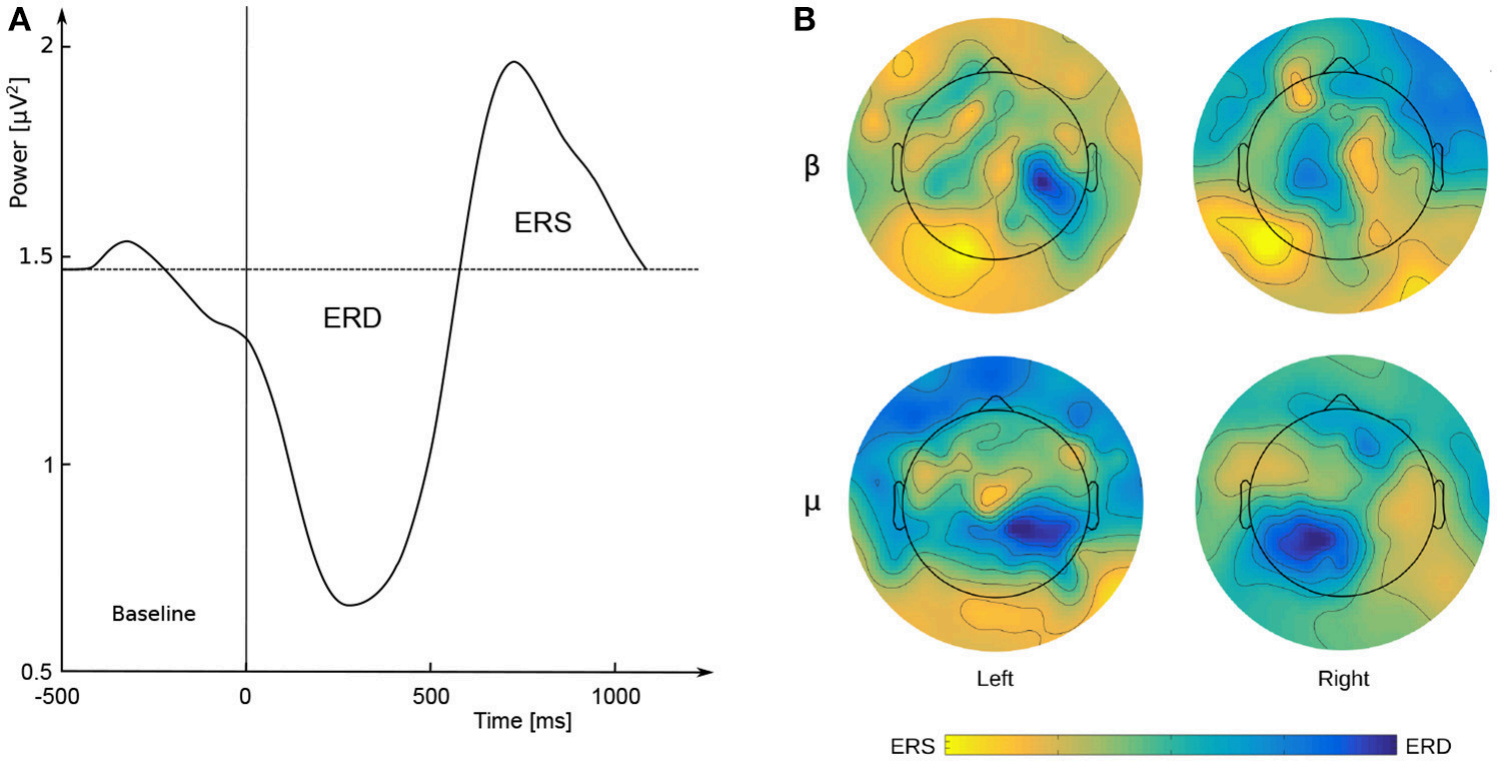
**(A)** Example of ERD/ERS time courses. The vertical line indicates the beginning of imagery movement. Source: prepared by the authors, based on Lemm et al. (2009), p. 3; **(B)** maps of signal strength (μ*V*^2^) distribution on the scalp (μ = 8–12 Hz; β = 18–30 Hz) during imagined movement of the left or right hand. Source: prepared by the authors.

Brain-Computer Interface (BCI) systems enable control of external software applications and devices without engaging any muscles and by only recording brain activity (Rak et al., 2012). BCIs transform the signal recorded with the use of various neuroimaging techniques into a response of external effectors. The process consists of a number of stages of data processing (Hwang et al., 2013). Electroencephalography (EEG) is the most frequently used neuroimaging method with a lot of possible applications still being postulated (Wojcik et al., 2018a,b) and for a medical purposes it can be supported by a variety of sophisticated tools offered by applied mathematics (Kakiashvili et al., 2012; Koczkodaj and Szybowski, 2015), cognitive science (Ogiela et al., 2008) or artificial intelligence (Szaleniec et al., 2013). The mechanisms most commonly used in contemporary BCIs include event related potentials (P300) (Sellers et al., 2012), Steady State Visual Evoked Potentials (SSVEP) (Byczuk et al., 2012; Kotyra and Wojcik, 2017a,b) and motor cortex activity (e.g., SMRBCI, Sensorimotor Rhythms BCI or MIBCI, Motor Imagery BCI) (Pfurtscheller and McFarland, 2012). Control with the use of MIBCI relies on the possibility of identifying brainwave patterns, measured with EEG from the scalp, during intended movement or imagined movement (e.g., clenching and relaxing one's hand) (Pineda, 2005). The process of MI is accompanied by a decrease in the power of Sensorimotor Rhythms (SMR) (in the range μ 8–12 Hz and β 18–30 Hz). The phenomenon is also known as Event-Related Desynchronisation (ERD) (Durka et al., 2001). On the other hand, after an imagined or actual movement is completed, there is an opposite process, i.e., an increase in the power of SMRs, otherwise known as Event-Related Synchronization (ERS) (Pfurtscheller, 1992). In the case of hand movements (whether imagined or real) the ERD/ERS effect occurs with varied intensity on the electrodes placed on both sides of a subject's head (e.g., C3 and C4). As a result, it is possible to determine e.g., whether the subject imagines movement of its right or its left hand due to the fact that ERD shows more strongly on the electrodes located contralaterally to the hand involved in the task (Pfurtscheller and Da Silva, 1999). The phenomenon of ERD/ERS for the imagined movement of the right and the left hand is presented in Figure 1.

If compared with other types of BCIs based on relatively automatic and passive physiological responses, like oscillations in voltage in visual cortex that are elicited by strobe light (SSVEP) or positive deflection elicited by events that present less-frequent than other events (P300), MIBCI is more similar to the way in which we control our environment with muscles. Translating the imagined movement into the reaction of a device or application is more natural and ergonomic than simply watching repeatedly highlighted objects on a screen. However, it is also less efficient and requires long-lasting individual training and calibration sessions (Friedrich et al., 2009). Nevertheless, there are many examples of possible applications of SMRBCI for various purposes, such as communication, videogames (Paszkiel, 2016), control of prosthetic devices (Pfurtscheller et al., 2000), control of rehabilitation equipment (Huang et al., 2012) and navigation in 2D and 3D space (Leeb et al., 2007). Control by means of changes in electrical activity of the brain is possible by the procedures employed in EEG signal processing. The specific operations to which the signal is subjected are designed to identify the characteristic changes in the continuous EEG recording (features extraction) and to match the fragments identified with specific mental activities (features classification) (Wolpaw and Wolpaw, 2012). Feature extraction and classification in MIBCI is carried out by the use of numerous algorithms, which differ in terms of effectiveness and are examined by many researchers (Bashashati et al., 2007; Lotte et al., 2007). The choice of an adequate signal processing procedure influences interface effectiveness. The brain activity associated with MI may have different characteristics from person to person, relative to anatomical or psychological factors (Ahn and Jun, 2015; Jeunet et al., 2015). Given the above, signal processing methods must take into account the individual variations of EEG activity in representation in space, time and frequency range.

During a BCI session, the recorded and pre-processed signal is subjected to further processing in order to identify those features representing the activity which will be used for interface control. Hence, information is extracted which is of significance for system operation, and the remaining data are rejected (Nicolas-Alonso and Gomez-Gil, 2012). MIBCIs may operate in synchronous and asynchronous ways. A user of synchronous BCI performs imagery tasks in strictly defined moments and for a specified duration of time. In the case of asynchronous BCIs, the moment and duration of the mental representation are unknown, and the analysis of the signal for the possible occurrence of imagery is a continuous process (Townsend et al., 2004).

The stage of features extraction is followed by conversion into the response of the device or computer application. This stage involves matching the previously extracted features with the state defined by the relevant type of BCI (Rak et al., 2012). BCI receives a time series representing the activity of the brain during imagery and information about the type of imagery (state), which has been performed. As a result of acquiring many pairs of such data, BCI can learn to match EEG data with appropriate states. During the trials carried out at the stage of testing, the interface is provided only with the data representing the activity of the brain. The interface is to correctly identify the imagery or its absence in the received input data. Based on the number of correctly identified imagery tasks, it is possible to calculate the effectiveness of the interface (CA). Classification algorithms most frequently use one of two approaches: classification by discrimination or classification by regression (McFarland et al., 2005). In classification by discrimination, the algorithm divides input data into independent groups, each of which corresponds to one of the states used to operate the interface. In classification by regression, the algorithm, taking into account the input data, calculates the value, which can then be arranged in order relative to the values computed for other input data. Determining the order this way enables the input data to be allocated to one of the states or translation of the brain activity into e.g., cursor movement on a computer screen (Wolpaw and Wolpaw, 2012). The regression and discrimination based classification models comprise a number of signal processing algorithms. These include such techniques as generative, linear, non-linear and combined techniques, i.e., using features of the previous methods (Nicolas-Alonso and Gomez-Gil, 2012). The basic property of all the classifiers is the effectiveness of classification, i.e., the efficiency with which they can translate the identified features into the desired response of the device (Wolpaw and Wolpaw, 2012). The effectiveness of classification is an indicator enabling comparison of various classification methods (Lotte et al., 2007). Cross validation is used to ensure the appropriate testing of classification effectiveness. In the course of the cross validation procedure, the set of all data acquired during the examination is subjected to multiple divisions into two subsets: the training and testing subsets. The classifier is taught to allocate input data to one of the states, based on the data in the training subset. The effects of the learning process are verified with the use of the testing subset. The mean effectiveness of classification for the test data in all of the divisions executed is a measure of CA.

### 1.2. Objectives and research questions

The main purpose of this review is to present the most popular methods in signal processing algorithms in MIBCI. It recapitulates the findings of reports presenting the effectiveness of MIBCI procedures by taking into account details related to data collection, preparation and analysis. Quantitative analysis was used to identify the most popular methods of data preprocessing, as well as extraction and classification of features significant for BCI control. The findings provided grounds for conclusions with regard to the dominating trends in research on signal processing in MIBCI. The trends observed were also examined for their consistency with developments and the methodological requirements postulated in the literature. Meta-analysis was performed in order to answer additional research questions: Question 1 (Q1) What is the average effectiveness of signal processing in the most popular types of MIBCI? Question 2 (Q2) Was there a significant increase in effectiveness of signal processing over the relevant period? Question 3 (Q3) Do methods of signal recording and processing (e.g., number of channels, spatial filtering, method of classification) influence the effectiveness of MIBCI control? Question 4 (Q4) Do the research findings show that the applied techniques enable signal classification which is sufficient for effective use of the interfaces? Based on the findings of descriptive analysis and meta-analysis, conclusions and recommendations were formulated. Suggestions were made for improvements with regard to changes in existing practices applied to research on signal processing in MIBCIs.

## 2. Materials and methods

### 2.1. Article selection

**Figure 2:**
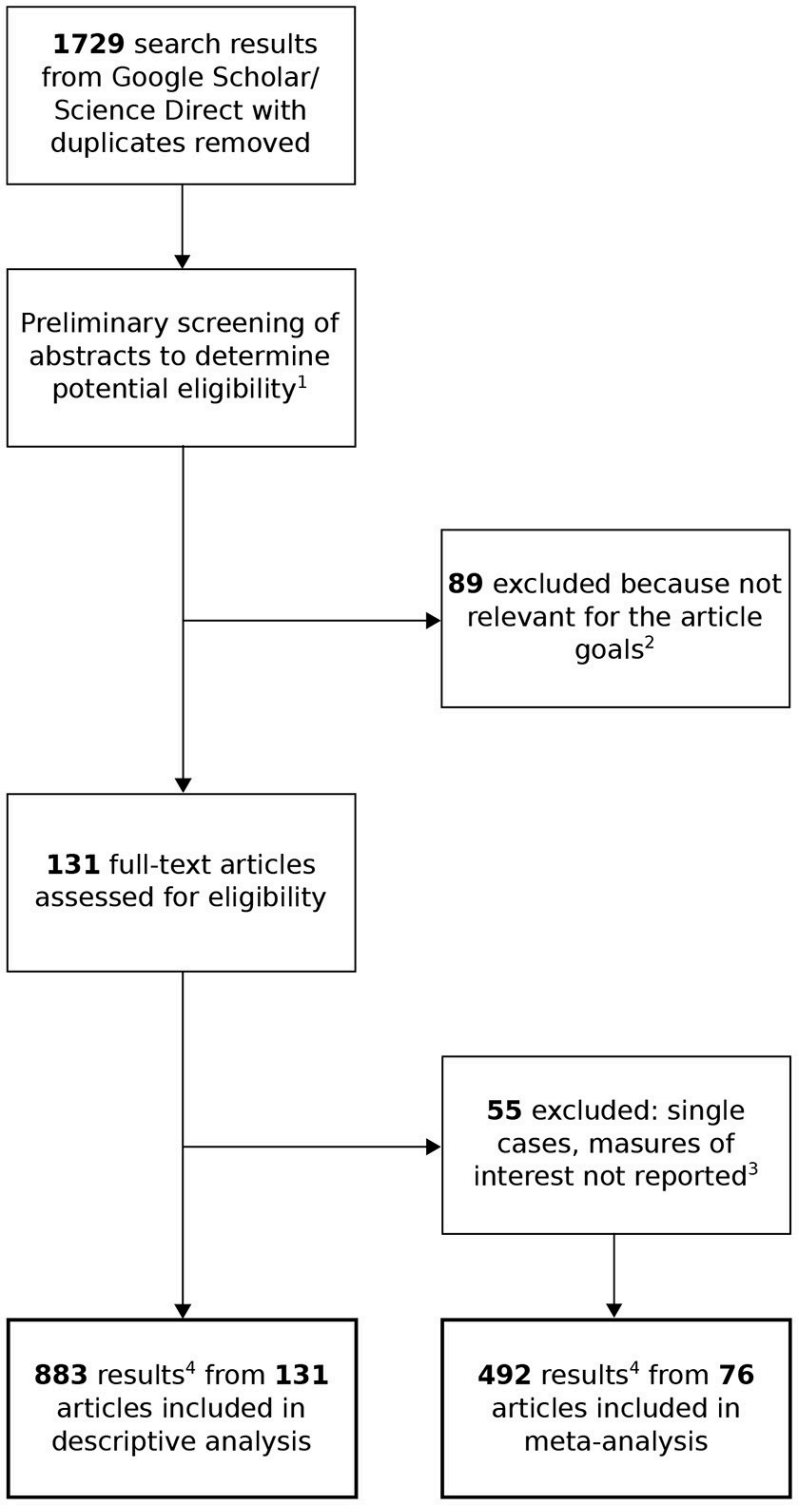
Flow diagram for identifying articles for the meta-analysis and descriptive analysis. (1) A total of 1,503 studies were excluded because of their irrelevance (e.g., reviews, gray literature, theoretical articles, reports from cognitive experiments), (2) based on criteria (studies without motor imagery condition; other than EEG analysis), (3) and which did not provide sufficient statistics, such as means, standard deviations and size, to drive meta-analysis. (4) A majority of the articles contained results of more than one analysis. Each variant of data processing was examined as a separate result/BCI design.

The review is based on 131 studies, published in English in peer-reviewed journals from 1997 to 2017. The reports were included in the review if they met all of the following criteria: (1) One or more of the keywords: motor imagery BCI, MI BCI, sensorimotor rhythms BCI, SMR BCI, Graz BCI, Wandsworth BCI, BCI Competition; (2) The reports described one or more BCI designs; (3) The reports providing sufficient data to estimate an effect size for meta-analysis (the studies that did not provide sufficient statistics were used in descriptive analysis). Supplementary approaches to identifying relevant studies included searching the references of a review article (Lotte et al., 2007). Articles were obtained through an online search of the ScienceDirect and Google Scholar databases. The contents of the articles were analyzed in order to identify the structural components of BCIs, which significantly impact the effectiveness of the systems. The analysis focused on procedures of data collection and data processing. The method of selecting articles is shown in Figure 2.

### 2.2. Data extraction

The following data were systematically extracted from each selected study: publication year, number of channels, information about dataset, number of subjects, mental task (number of categories), pre-processing (i.e., manual or automatic rejections of artifacts), feature extraction method, feature classification method, average classification accuracy (CA), standard deviation of CA and information enabling calculation of level of chance (LC) performance. LC is defined as the level of CA that can be reached by chance: LC = 100%/number of targets (Marchetti and Priftis, 2015). Due to the varied number of categories (mental imagery) applied in the specific studies CA was transformed into values corrected with the LC in a given type of interface, as proposed by Marchetti and Priftis (2015) Corrected CA (CCA) = (CA − LC) ^*^ 100/(100 − LC). The CCA value was used as the classification effectiveness rate in some of the descriptive analyses and in meta-analysis.

### 2.3. Statistical analyses

Quantitative analysis was based on descriptive statistics, taking into account percent distribution, number, and modal values pertaining to the aspects of signal processing in MIBCI investigated. The results from studies which contained information sufficient for calculating the effect size were included in the meta-analysis. The approach to random-effects adopted for the calculations reflected the assumption that the effect sizes of the studies represented a random sample of the real ones (Borenstein et al., 2011; Cumming, 2013; Marchetti and Priftis, 2015). The heterogeneity and inconsistency among studies were obtained with the Cochran's Q and I^2^ tests (Higgins et al., 2003). To address possible publication bias, Egger's regression tests were computed (Rothstein et al., 2006). The average performance level of the most popular signal processing methods (Q1) has been calculated (CCA), with confidence intervals (95%). Publication year (Q2) and numbers of channels (Q3) were used as moderator variables in meta-regression to test the effects of publication time and sampling density on CCA. Inverse Variance Weighted ANOVA was used to test the differences across mean CCA for categorical variables (Q3), such as: numbers of targets (2, 3, or 4 categories), spatial filtering (CSP or band power), classification algorithms (DA or SVM) and evaluation methods (online or offline). In order to address the problem contained in Q4, a *t*-test was performed by comparing CA with LC. Analyses and their graphic visualizations were prepared using the “metagen” (v. 1.0) package for statistical software R (v. 3.3.2) and IBM SPSS Statatistic PL (v. 21).

## 3. Results

### 3.1. Descriptive analysis

The operation of each EEG-BCI system starts with recording the bioelectric activity of the brain with the use of electrodes. From the point of view of BCIs, the critically important features include the system's tolerance to distortions in EEG recording and the ergonomics of using the device. Unlike the EEG systems used in e.g., performing medical diagnoses or psycho-physiological experiments, the equipment employed for the specific needs of BCI must be easy to operate and maintain, must enable long-lasting operation without a decrease in the accuracy of the recording and must be relatively cheap. This is because BCIs are mainly intended for individuals with disabilities using such devices at home, and this is associated with a greater risk of artifacts. To meet the related requirements, EEG-BCI systems generally utilize a lower number of electrodes than clinical EEGs. By using less than 20 downleads, it is possible to reduce the costs of the device and enable rapid application (Portelli et al., 2011).

The number of channels used in the studies currently being conducted shows significant variation (Table 1). No evidence has been found for the use of records from high-density EEG nets, which comprise over 128 channels. The articles in the review suggest that the most frequently used number of channels ranges from 3 to 8 (21.61% of cases) and from 1 to 2 (20.33% of cases).

**Table 1:** Number of channels in the record analyzed.

**Number of channels**	**Percentage of studies**
1–2	20.33
3–8	21.61
9–16	10.05
17–31	19.16
32–64	14.49
65–128	14.37

SMRBCIs are active BCIs; they take advantage of the changes in the recording of brain activity in response to the mental task which is being performed. A number of different tasks are executed by users of MIBCI to control the device. These include: imagined movement vs. relaxing (Neuper et al., 2003), imagined right hand vs. left hand movement (Scherer et al., 2009) and imagined movement of the foot or tongue (Leeb et al., 2008). While evaluating specific types of mental tasks applied in MIBCI, it is necessary to take into account the number of decisions, which can be made using a given procedure. The systems involving imagined movement vs. relaxing and right vs. left hand movement enable a choice to be made between two conditions (0 and 1), which is sufficient e.g., for virtual keyboard control but not for movement in three-dimensional space (Wolpaw and Wolpaw, 2012). The effectiveness of recognition of MI in BCI in the articles reviewed was most frequently tested with the use of a signal representing two states (76.70% cases), imagined right and left hand movement being the predominant procedure. Recordings, which represent three states, such as the imagined movement of both hands and the tongue, are considerably less common (Table 2). 97% of studies used synchronous control, while the others used asynchronous.

**Table 2:** Types of imagery used in algorithm testing.

**Types of imagery**	**Percentage of studies**
Left hand, right hand	53.51
Right hand, right foot	9.05
Left hand, right hand, foot	8.48
Right hand, foot	2.60
Other	26.36

All of the reviewed articles present results acquired with the use of more than one signal processing algorithm. They compare a number of combinations of extractors, selectors and classifiers tested with the same datasets. The selected reports contained findings of 883 experiments, yet only 4.90% of the total number of experiments were related to studies in which algorithms were tested online, i.e., during real-time interface control. In the remaining cases, signal processing was conducted based on datasets of EEG recordings performed during MI tasks. The majority of the latter group used the signals available in the framework of the BCI Competitions (Brunner et al., 2008; Leeb et al., 2008). This is a project which has provided a large group of researchers with access to EEG datasets in order to facilitate the comparison of various signal processing techniques and their effectiveness. In the selected articles, 78.90% of the results were based on data from BCI Competitions. The studies differed regarding the number of subjects. Most frequently, their number was in the range of *N* = 2–10 (75.47% of cases). A significant group represented the findings of case studies, i.e., involving one subject only (21.11%). Few analyses were carried out for study groups of more than 10 subjects (3.42% of cases). The most frequently applied practice at the stage of data pre-processing involves band-pass filtering of the signal. As a result, the signal only retains those frequency bands which represent the electrical activity of the brain associated with MI (in most cases: μ (8–12 Hz) and β (18–30 Hz). Generally, the data designated for analyses were not subjected to the procedure of removing artifacts from the signal, yet, in some cases, the recording was purified manually (11.31%).

A number of signal processing methods are employed at the stage of the extraction of features (Lotte et al., 2007). Features within the EEG signal are most frequently determined with variants of the Common Spatial Patterns method (45.83% cases), which, in terms of popularity, is followed by band power (17.88% cases). It is worth to note that 15 studies using CSP conducted the analyses on fewer channels than recommended minimum of 8 for this method. The publications reviewed most frequently used signal classification via linear methods based on such algorithms as Discriminant Analysis (DA) (38.14% of cases) and Support Vector Machine (SVM) (33.84% of cases).

### 3.2. Meta-analysis

**Figure 3:**
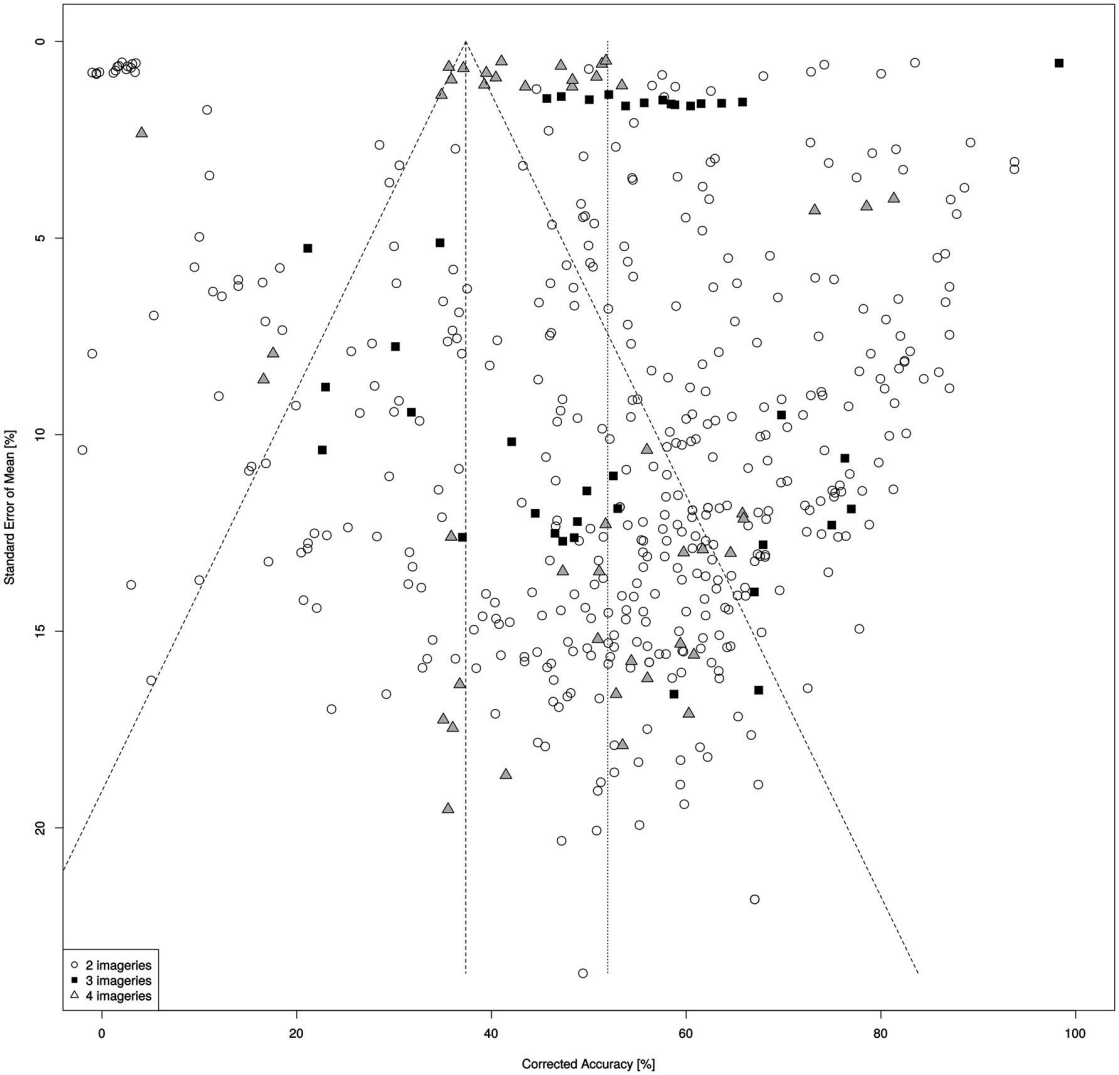
Funnel plot for different types of MIBCI.

CCA in the studies included in the meta-analysis is 51.96% (CI 95% = 50.07 to 53.86), 53.24% for synchronous and 22.71% for asynchronous tests. The results are characterized by significant heterogeneity (*Q* = 1806577.61, *df* = 486; *p* < 0.001; *I*^2^ = 99.97%) and the effects of publication bias (Egger's test *t* = 152,41, *p* < 0.001), shown in a funnel plot (Figure 3).

Meta-regression with the number of channels used in the analyses and with publication year showed a significant impact of the two moderator variables in CCA (*Qmodel* = 98.05, *p* < 0.001). An increase in the number of channels coincided with greater CCA (number of channels; *B* = 0.09, CI 95%: = 0.07 to 0.12; *p* < 0.001). On the other hand no correspondence was observed between the reported CCA and the year in which the results were published. In fact, during the period in question the accuracy of the solutions examined decreased significantly (year; *B* = −0.97, CI 95%: −1.26 to −0.68; *p* = 0.005). Inverse Variance Weighted Oneway ANOVA was applied to examine the effect of the number of targets (2, 3, or 4 categories) on the homogeneity of distribution (*Qbetween* = 7.36, *df* = 2; *p* = 0.02). In a two-category design CCA = 52.12% (CI 95%: = 50.96 to 53.81), and is characterized by a significant heterogeneity of results (*Qw* = 1299.47, *df* = 399; *p* < 0.001; *I*^2^ = 69.36%). For three mental images the mean result was 53.56% (CI 95%: = 49.81 to 57.31) with a high degree of heterogeneity of results (*Qw* = 72.16, *df* = 37; *p* < 0.001; *I*^2^ = 50.11%). In the case of four categories the result was 47.66% (CI 95%: = 44.41 to 50.91) and also was characterized by a high level of heterogeneity in the distribution (*Qw* = 79.78, *df* = 48; *p* = 0.002; *I*^2^ = 41.09%). A separate analysis examined the effect of spatial filtering of signal (CSP vs. Band Power), which was shown to significantly impact the distribution of the results (*Qbetween* = 32.02, *df* = 1; *p* < 0.001). CCA in studies which applied CSP was found at the level of 53.5% (CI 95%: = 51.69 to 55.3), with significant heterogeneity (*Qw* = 514.15, *df* = 231; *p* < 0.001; *I*^2^ = 55.27%). In the cases applied band power CCA was at the level of 43.66% (CI 95%: = 40.77 to 46.55), also with a heterogeneous distribution (*Qw* = 139.03, *df* = 87; *p* < 0.001; *I*^2^ = 38.14%). The method of evaluation of the results (offline vs. online) has no significant influence on the CCA (*Qbetween* = 2.04, *df* = 1; *p* = 0.153). Also, the two most popular methods of signal classification (DA and SVM) did not differ in terms of their impact on the homogeneity of the effects obtained (*Qbetween* = 0.29, *df* = 1; *p* = 0.592). CA in each of the types of MIBCI examined was significantly higher than LC: two categories [*t*_(400)_ = 44.29, *p* < 0.001]; three categories [*t*_(38)_ = 20.05, *p* < 0.001] and four categories [*t*_(49)_ = 22.75, *p* < 0.001]. Nevertheless, CCA higher than the 60% threshold rate for effective control (Guger et al., 2003) was achieved in 37% of the studies and the result exceeded 70% (Brunner et al., 2010) in only 16.6% of the studies. It should be noted that in the group of 14 studies analysing real-time BCI control, CCA was below the threshold value of 60% in four cases (28.6%) although the CCA in this group of results still differed from LC [*t*_(13)_ = 8.13, *p* < 0.001].

## 4. Discussion

Since the last decade of the twentieth century and in the beginning of the twenty first century there has been growing interest in issues related to BCIs (Huggins and Wolpaw, 2014). As pointed out by researchers, studies into BCI mainly focus on improving the effectiveness of signal processing algorithms (Hwang et al., 2013). Nevertheless, the effectiveness of information transfer aided by BCIs is insufficient for the widespread practical application of the technique (Ahn and Jun, 2015). Because of the easy application and price, the most common BCIs today are EEG based, and, in this group, the most common are those in which control is based on MI (Hwang et al., 2013). This explains the large number of articles focusing on the classification of signals containing MI. Yet, it is unclear to what extent findings and conclusions from these studies can be utilized in practice for engineering more effective SMRBCI systems.

### 4.1. Quality of study

In accordance with the “gold standard” proposed for BCI-related research by McFarland and Krusienski (Wolpaw and Wolpaw, 2012), the most valuable results showing possible uses of a given algorithm in an existing interface are acquired by means of both offline and online studies. This means that the new method is subjected to testing with the use of generally accessible models and readily available sets of records, as well as during sessions where users control the interface in real time. This approach makes it possible to compare the findings of a specific study with the results acquired with other techniques and to answer the question as to whether a given method is effective if utilized in a real-life situation. Obviously, this approach is much more challenging and labor-intensive, as reflected in the significantly lower number of publications containing accounts of online experiments (4.9% of cases). The majority of the remaining studies contain analyses carried out with the use of datasets from BCI Competitions (Blankertz et al., 2010). This can be recognized as an advantage since the results of various experiments based on the same datasets are easier to compare; on the other hand, the possibility of translating the results into BCI operation in real-life settings is significantly limited. Results of the meta-analysis show that the CCA differed depending on whether the algorithm was assessed offline or online. Given this, the solutions subjected to testing based exclusively on cross-validation may be overestimated with regard to their effectiveness in comparison to real-time application of BCI in communication. The findings acquired during online analyses were more homogeneous which may suggest that irrespective of the method applied, participants in the studies controlled the interface with similar effectiveness. The fact that the relevant analyses are based on an insufficient number of observations is a drawback from the viewpoint of the practical applicability of the findings related to algorithms of signal processing in MIBCI. Proportionally, a significant number of experiments are based on datasets acquired from one subject, and analyses based on groups of more than 10 subjects are a rarity. It is a well-established fact that subject-specific differences significantly impact the effectiveness of BCI control (Blankertz et al., 2010; Hammer et al., 2012). According to some researchers, a considerable number of users, from several to a few dozen percent, face significant difficulties in trying to effectively convey information in this way (Allison and Neuper, 2010). This phenomenon is sometimes called BCI illiteracy or aphasia (Millán et al., 2010). Additionally, there are differences among subjects reflecting the varied pace and effectiveness of learning to control BCIs (McFarland et al., 2005). Differences of this kind occurring in the population may impact the findings of analyses related to small study groups. The applicability of the reported findings may also be adversely affected by errors occurring at the stage of data preparation and during the presentation of results. Although MIBCIs are designed for real-time operation, some studies are based on records manually purged of artifacts (11% of cases). The procedure of removing fragments of a signal contaminated with non-representative brain activity, e.g., caused by eye or muscle movements, is widely used in EEG based studies (Jung et al., 2000). The method, however, cannot be introduced during the real-time process of BCI control with minimum operation by people other than the user. The effectiveness of the algorithm in recognizing signal patterns in an initially purified recording may lead to inflating the final result and may fail to represent the actual conditions of BCI operation. In order to ensure comparability of results showing the effectiveness of signal processing achieved by various methods, it is necessary to adopt a similar way of reporting results. Researchers, however, use various ways of presenting the findings; for instance, they report the mean error in signal classification, the mean percentage of correctly classified samples or results related to the most successfully or least effectively classified fragments of data. More than half of the reviewed articles could not be included in the meta-analysis due to the lack of information necessary for estimating the effect size (see Figure 2). Such a method of conducting analyses and reporting the results makes it impossible to compare the findings acquired by different researchers. The matter is additionally confounded by the fact that MIBCIs differ in terms of the number of imagery tasks performed by the user to control the system (e.g., imagined right hand and left hand movement or imagined movement of the hand and foot), or they vary in terms of the length of a single trial. Hence, the same result related to various types of BCIs does not represent the same effectiveness. Due to this, some researchers have postulated a method of converting the results into the number of bits of information that can be transmitted by the relevant BCI per a specified unit of time e.g., minute (McFarland et al., 2003). This method of converting the results is not commonly used in the articles reviewed. Some articles contain insufficient information related to the method of recording and preparing the data for analyses. Examples of practices which seem inadequate include: lack of information about a reference channel or the impedance value, presentation of the results exclusively in graphical form without providing numerical values, and an interpretation of differences between algorithms without subjecting them to statistical testing. Improvements in these standards may contribute to an increased scientific value and higher applicability of research findings related to data processing in MIBCI. In order to unify the method of reporting the most important aspects of the research, the authors suggest that articles should contain a table with a summary of the study (Table 3). It would enable quick assessment of experimental findings and would facilitate access to important data from the viewpoint of future replication studies or practical application. Moreover, the concise presentation of data related to equipment and analysis would leave more space in the article for information about the procedure and mathematical basics underlying the method of applied signal processing. The proposed chart is divided into five parts: 1: Settings, 2: Data set, 3: Study group, 4: Procedure and 5: Results. The first, second, and third parts contain information on the method of data acquisition. The fourth part relates to details of the algorithms tested. The final section presents the findings. The shape and contents of the chart can be freely modified. The tool is, in fact, a point of reference for the concise presentation of information regarding the method of analysing data in a BCI experiment.

**Table 3:** The sample design of table that could be used to summarize relevant information about a study.

**Settings**		
Amplifier model		
Cap model		
Type of electrodes		
Recorded channels [N]		
Analyzed channels [N]		
Reference		
Ground		
Impedance		
**Data set**		
Name		
Source		
**Study group**		
Subjects [N]		
Males [N]		
Females [N]		
Right-handed [N]		
Healthy [N]		
Experienced [N]		
Age (Avg)		
Age (SD)		
**Procedure**		
Motor imagery task description		
Trials [N]		
Trial duration [s]		
Synchronous [Y/N]		
On-line [Y/N]		
	**Methods**	**References**
Pre-processing		
Feature extraction		
Feature selection		
Feature classification		
**Results**		
Accuracy (Avg) [%]		
Accuracy (SD) [%]		
ITR [bps]		

### 4.2. Signal processing methods

The findings of meta-regression suggest that the number of channels taken into account in a study corresponds with a higher CCA rate. Indeed, this variable should be taken into account in future studies, e.g., by testing the same algorithms with the use of data for low and high recording density. On the other hand, by decreasing the number of channels in BCI it would be possible to reduce the cost of such devices, which would make BCIs more accessible (Portelli et al., 2011). The use of spatial filtering (e.g., common spatial patterns) affects results achieved at the stage of classification. In studies which applied only band power for the extraction of signal features, CCA was significantly lower than the results achieved with spatial filtering. This is consistent with research findings suggesting that the spatial representation of the ERD/ERS effect differs depending on individual factors, such as motor experience (Zapała et al., 2015), mental imagery capacities (Marchesotti et al., 2016) or lateralization (Stancák and Pfurtscheller, 1996). The most popular signal classifiers (DA and SVM) did not impact the effects reported in the articles reviewed. Operation of the two methods is relatively similar and involves discrimination of signal samples. There were too few experiments based on alternative methods of feature translation, such as regression or artificial neural networks, so they could not be included in the meta-analysis.

### 4.3. Effectiveness of MIBCI control

CA in all the main types of MIBCI is significantly higher than LC. However, the level of classification recognized as sufficient for effective communication is only achieved by some of the reported systems of signal processing. Taking this into account we can say that in the case of a significant majority of studies, if classification took place in a similar way as during offline assessment, the BCI users would have problems with effective communication by means of the device. More importantly, the results of offline evaluations, which in this review constitute a majority, still may be overestimated in comparison to the effects achieved during real-time control (Wolpaw and Wolpaw, 2012). The findings of the meta-regression, with the publication year as a moderator, do not permit the conclusion that there was an improvement in the effectiveness of signal classification during the relevant period. In fact the significantly negative result of the regressions suggests an opposite trend. This may suggest that the systems currently used are ineffective. More and more studies point to the relationship between intra- and inter-subject factors and the operation of BCIs (Ahn and Jun, 2015) and refer to the necessity of applying training enhancing the vividness of MI in users of such systems (Lotte and Jeunet, 2015). Perhaps the architecture of MIBCI systems is in need of greater change than those which have so far been tested.

### 4.4. Heterogeneity and publication bias

Lack of homogeneity in the case of the data examined may result from the use of different, and sometimes incomparable, signal processing procedures. The significant effects of such moderators as: number of channels, spatial filtering or number of mental images show that these are important variables which should be controlled in future experiments. The publication bias identified by Egger's test may be interpreted as a tendency to mainly report results exceeding LC. This does not greatly impact the reliability of the effects that are achieved because the postulated levels sufficient for effective use of BCI are estimated at a higher level than 50%. Algorithms with an CCA exceeding 60 or 70% still constitute a minority in reported studies.

### 4.5. Conclusions

By reference to the questions which were asked at the start, it can be assumed that signal processing algorithms applied in MIBCI enable signal classification at a rate significantly exceeding LC (Q1; Q4). On the other hand, only a small fraction of these achieve results recognized as sufficient to ensure that actual operation of interfaces is effective. High heterogeneity of results and the lack of an effect showing an increase in algorithm efficiency over time (Q2) suggest that there are a number of variables potentially affecting the ultimate classification result, yet they are not sufficiently controlled in experimental procedures (Q3). Such factors may include those connected with signal processing (e.g., number of channels applied, spatial filtering of the signal, and type of mental imagery) as well as individual differences whose relationship with BCI operation is confirmed by research (Ahn and Jun, 2015). The poor quality of the published findings is a considerable impediment from the point of view of MIBCI development. Many of the selected articles did not present key information enabling a comparison to be made with other studies. Without a standardized method of conducting such experiments and reporting their results, it will be impossible to use such algorithms in practice. To improve the situation the following is recommended: (1) Algorithms should be tested both online and offline. (2) Reports should provide sufficient information to replicate a study and perform meta-analysis. (3) Key features of a study should be summarized in a clear and possibly uniform manner (for instance: Table 3) (4) The design of BCI data processing should consist of steps which are applicable in real-time BCI control (e.g., without manually removed artifacts).

## Author contributions

All authors listed have made a substantial, direct and intellectual contribution to the work, and approved it for publication.

### Conflict of interest statement

The authors declare that the research was conducted in the absence of any commercial or financial relationships that could be construed as a potential conflict of interest.

## References

[B1] Ahn M. Jun S. C. (2015). Performance variation in motor imagery brain–computer interface: a brief review. J. Neurosci. Methods 243, 103–110. 10.1016/j.jneumeth.2015.01.033 25668430

[B2] Allison B. Z. Neuper C. (2010). Could anyone use a BCI?, in Brain-Computer Interfaces, Human-Computer Interaction Series, eds Tan D. Nijholt A. (London: Springer), 35–54.

[B3] Bashashati A. Fatourechi M. Ward R. K. Birch G. E. (2007). A survey of signal processing algorithms in brain–computer interfaces based on electrical brain signals. J. Neural Eng. 4:R32. 10.1088/1741-2560/4/2/R03 17409474

[B4] Blankertz B. Sannelli C. Halder S. Hammer E. M. Kübler A. Müller K. R.. (2010). Neurophysiological predictor of SMR-based BCI performance. Neuroimage 51, 1303–1309. 10.1016/j.neuroimage.2010.03.022 20303409

[B5] Borenstein M. Hedges L. V. Higgins J. P. Rothstein H. R. (2011). Introduction to Meta-Analysis. Chichester: John Wiley & Sons.

[B6] Brunner C. Allison B. Z. Krusienski D. J. Kaiser V. Müller-Putz G. R. Pfurtscheller G.. (2010). Improved signal processing approaches in an offline simulation of a hybrid brain–computer interface. J. Neurosci. Methods 188, 165–173. 10.1016/j.jneumeth.2010.02.002 20153371 PMC3422070

[B7] Brunner C. Leeb R. Müller-Putz G. Schlögl A. Pfurtscheller G. (2008). Bci Competition 2008-Graz Data Set a. Graz: Institute for Knowledge Discovery; Laboratory of Brain-Computer Interfaces; Graz University of Technology.

[B8] Byczuk M. Poryzała P. Materka A. (2012). SSVEP-based brain-computer interface: on the effect of stimulus parameters on VEPs spectral characteristics, in Human-Computer Systems Interaction: Backgrounds and Applications 2. Advances in Intelligent and Soft Computing, eds Hippe Z. S. Kulikowski J. L. Mroczek T. (Berlin; Heidelberg: Springer), 3–14.

[B9] Cumming G. (2013). Understanding the New Statistics: Effect Sizes, Confidence Intervals, and Meta-analysis. New York, NY: Routledge.

[B10] Durka P. J. Ircha D. Neuper C. Pfurtscheller G. (2001). Time-frequency microstructure of event-related electro-encephalogram desynchronisation and synchronisation. Med. Biol. Eng. Comput. 39, 315–321. 10.1007/BF02345286 11465886

[B11] Friedrich E. V. McFarland D. J. Neuper C. Vaughan T. M. Brunner P. Wolpaw J. R. (2009). A scanning protocol for a sensorimotor rhythm-based brain–computer interface. Biol. Psychol. 80, 169–175. 10.1016/j.biopsycho.2008.08.004 18786603 PMC2952890

[B12] Guger C. Edlinger G. Harkam W. Niedermayer I. Pfurtscheller G. (2003). How many people are able to operate an EEG-based brain-computer interface (BCI)? IEEE Trans. Neural Syst. Rehabil. Eng. 11, 145–147. 10.1109/TNSRE.2003.814481 12899258

[B13] Hammer E. M. Halder S. Blankertz B. Sannelli C. Dickhaus T. Kleih S.. (2012). Psychological predictors of SMR-BCI performance. Biol. Psychol. 89, 80–86. 10.1016/j.biopsycho.2011.09.006 21964375

[B14] Higgins J. P. Thompson S. G. Deeks J. J. Altman D. G. (2003). Measuring inconsistency in meta-analyses. Brit. Med. J. 327:557. 10.1136/bmj.327.7414.557 12958120 PMC192859

[B15] Huang D. Qian K. Fei D. Y. Jia W. Chen X. Bai O. (2012). Electroencephalography (EEG)-based brain–computer interface (BCI): a 2-d virtual wheelchair control based on event-related desynchronization/synchronization and state control. IEEE Trans. Neural Syst. Rehabil. Eng. 20, 379–388. 10.1109/TNSRE.2012.2190299 22498703

[B16] Huggins J. E. Wolpaw J. R. (2014). Papers from the fifth international brain–computer interface meeting. J. Neural Eng. 11:030301. 10.1088/1741-2560/11/3/030301 24837824 PMC4438077

[B17] Hwang H.-J. Kim S. Choi S. Im C.-H. (2013). Eeg-based brain-computer interfaces: a thorough literature survey. Int. J. Hum. Comput. Interact. 29, 814–826. 10.1080/10447318.2013.780869

[B18] Jeunet C. N'Kaoua B. Subramanian S. Hachet M. Lotte F. (2015). Predicting mental imagery-based bci performance from personality, cognitive profile and neurophysiological patterns. PLoS ONE 10:e0143962. 10.1371/journal.pone.0143962 26625261 PMC4666487

[B19] Jung T. P. Makeig S. Humphries C. Lee T. W. Mckeown M. J. Iragui V.. (2000). Removing electroencephalographic artifacts by blind source separation. Psychophysiology 37, 163–178. 10.1111/1469-8986.3720163 10731767

[B20] Kakiashvili T. Koczkodaj W. W. Woodbury-Smith M. (2012). Improving the medical scale predictability by the pairwise comparisons method: evidence from a clinical data study. Comput. Methods Prog. Biomed. 105, 210–216. 10.1016/j.cmpb.2011.09.011 22088867

[B21] Koczkodaj W. W. Szybowski J. (2015). Pairwise comparisons simplified. Appl. Math. Comput. 253, 387–394. 10.1016/j.amc.2014.12.069

[B22] Kotyra S. Wojcik G. M. (2017a). The station for neurofeedback phenomenon research, in Polish Conference on Biocybernetics and Biomedical Engineering (Cham: Springer), 32–43.

[B23] Kotyra S. Wojcik G. M. (2017b). Steady state visually evoked potentials and their analysis with graphical and acoustic transformation, in Polish Conference on Biocybernetics and Biomedical Engineering (Cham: Springer), 22–31.

[B24] Leeb R. Brunner C. Müller-Putz G. Schlögl A. Pfurtscheller G. (2008). BCI Competition 2008–Graz Data Set b. Graz: Graz University of Technology.

[B25] Leeb R. Lee F. Keinrath C. Scherer R. Bischof H. Pfurtscheller G. (2007). Brain–computer communication: motivation, aim, and impact of exploring a virtual apartment. IEEE Trans. Neural Syst. Rehabil. Eng. 15, 473–482. 10.1109/TNSRE.2007.906956 18198704

[B26] Lemm S. Müller K. R. Curio G. (2009). A generalized framework for quantifying the dynamics of EEG event-related desynchronization. PLoS Comput. Biol. 5:e1000453. 10.1371/journal.pcbi.1000453 19662156 PMC2713829

[B27] Lotte F. Congedo M. Lécuyer A. Lamarche F. Arnaldi B. (2007). A review of classification algorithms for EEG-based brain–computer interfaces. J. Neural Eng. 4:R1. 10.1088/1741-2560/4/2/R01 17409472

[B28] Lotte F. Jeunet C. (2015). Towards improved BCI based on human learning principles, in 2015 3rd International Winter Conference on Brain-Computer Interface (BCI) (Sabuk), 1–4.

[B29] Marchesotti S. Bassolino M. Serino A. Bleuler H. Blanke O. (2016). Quantifying the role of motor imagery in brain-machine interfaces. Sci. Rep. 6:24076. 10.1038/srep24076 27052520 PMC4823701

[B30] Marchetti M. Priftis K. (2015). Brain–computer interfaces in amyotrophic lateral sclerosis: a metanalysis. Clin. Neurophysiol. 126, 1255–1263. 10.1016/j.clinph.2014.09.017 25449558

[B31] McFarland D. J. Sarnacki W. A. Vaughan T. M. Wolpaw J. R. (2005). Brain-computer interface (bci) operation: signal and noise during early training sessions. Clin. Neurophysiol. 116, 56–62. 10.1016/j.clinph.2004.07.004 15589184

[B32] McFarland D. J. Sarnacki W. A. Wolpaw J. R. (2003). Brain–computer interface (bci) operation: optimizing information transfer rates. Biol. Psychol. 63, 237–251. 10.1016/S0301-0511(03)00073-5 12853169

[B33] Millán J. D. R. Rupp R. Mueller-Putz G. R. Murray-Smith R. Giugliemma C. Tangermann M.. (2010). Combining brain–computer interfaces and assistive technologies: state-of-the-art and challenges. Front. Neurosci. 4:161. 10.3389/fnins.2010.00161 20877434 PMC2944670

[B34] Neuper C. Müller G. R. Kübler A. Birbaumer N. Pfurtscheller G. (2003). Clinical application of an eeg-based brain–computer interface: a case study in a patient with severe motor impairment. Clin. Neurophysiol. 114, 399–409. 10.1016/S1388-2457(02)00387-5 12705420

[B35] Nicolas-Alonso L. F. Gomez-Gil J. (2012). Brain computer interfaces, a review. Sensors 12, 1211–1279. 10.3390/s120201211 22438708 PMC3304110

[B36] Ogiela L. Tadeusiewicz R. Ogiela M. R. (2008). Cognitive techniques in medical information systems. Comput. Biol. Med. 38, 501–507. 10.1016/j.compbiomed.2008.01.017 18339366

[B37] Paszkiel S. (2016). Control based on brain-computer interface technology for video-gaming with virtual reality techniques. J. Automat. Mobile Robot. Intell. Syst. 10, 3–7. 10.14313/JAMRIS_4-2016/26

[B38] Pfurtscheller G. (1992). Event-related synchronization (ERS): an electrophysiological correlate of cortical areas at rest. Clin. Neurophysiol. 83, 62–69. 10.1016/0013-4694(92)90133-3 1376667

[B39] Pfurtscheller G. Guger C. Müller G. Krausz G. Neuper C. (2000). Brain oscillations control hand orthosis in a tetraplegic. Neurosci. Lett. 292, 211–214. 10.1016/S0304-3940(00)01471-3 11018314

[B40] Pfurtscheller G. Lopes da Silva F. H. (1999). Event-related EEG/MEG synchronization and desynchronization: basic principles. Clin. Neurophysiol. 110, 1842–1857. 10.1016/S1388-2457(99)00141-8 10576479

[B41] Pfurtscheller G. McFarland D. J. (2012). BCIa that use sensorimotor rhythms, in Brain-Computer Interfaces: Principles and Practice, eds Wolpaw J. Wolpaw E. W. (New York, NY: Oxford University Press), 227–240.

[B42] Pineda J. A. (2005). The functional significance of mu rhythms: translating “seeing” and “hearing” into “doing. Brain Res. Rev. 50, 57–68. 10.1016/j.brainresrev.2005.04.005 15925412

[B43] Portelli A. J. Daly I. Spencer M. Nasuto S. J. (2011). Low cost brain-computer interface: first results, in Proceedings of the 5th International Brain-Computer Interface Conference 2011, eds, Müller-Putz G. R. Scherer R. Billinger M. Kreilinger A. Kaiser V. Neuper C. (Graz: Verlag der Technischen Universität Graz 2011; Graz University of Technology), 320–323.

[B44] Rak R. J. Kołodziej M. Majkowski A. (2012). Brain-computer interface as measurement and control system the review paper. Metrol. Meas. Syst. 19, 427–444. 10.2478/v10178-012-0037-4

[B45] Rothstein H. R. Sutton A. J. Borenstein M. (2006). Publication Bias in Meta-Analysis: Prevention, Assessment and Adjustments. Chichester: John Wiley & Sons.

[B46] Scherer R. Müller-Putz G. R. Pfurtscheller G. (2009). Flexibility and practicality: graz brain–computer interface approach. Int. Rev. Neurobiol. 86, 119–131. 10.1016/S0074-7742(09)86009-1 19607995

[B47] Sellers E. W. Arbel Y. Donchin E. (2012). BCIs that use p300 event-related potentials, in Brain-Computer Interfaces: Principles and Practice, eds Wolpaw J. Wolpaw E. W. (New York, NY: Oxford University Press), 215–226.

[B48] Stancák A. Jr. Pfurtscheller G. (1996). Event-related desynchronisation of central beta-rhythms during brisk and slow self-paced finger movements of dominant and nondominant hand. Cogn. Brain Res. 4, 171–183. 10.1016/S0926-6410(96)00031-6 8924046

[B49] Szaleniec J. Wiatr M. Szaleniec M. SkłAdzień J. Tomik J. Oleś K.. (2013). Artificial neural network modelling of the results of tympanoplasty in chronic suppurative otitis media patients. Comput. Biol. Med. 43, 16–22. 10.1016/j.compbiomed.2012.10.003 23174627

[B50] Townsend G. Graimann B. Pfurtscheller G. (2004). Continuous EEG classification during motor imagery-simulation of an asynchronous BCI. IEEE Trans. Neural Syst. Rehabil. Eng. 12, 258–265. 10.1109/TNSRE.2004.827220 15218939

[B51] Wojcik G. M. Masiak J. Kawiak A. Kwasniewicz L. Schneider P. Polak N. (2018b). Mapping the human brain in frequency band analysis of brain cortex electroencephalographic activity for selected psychiatric disorders. Front. Neuroinformatics 12:73 10.3389/fninf.2018.00073 PMC6207640 30405386

[B52] Wojcik G. M. Masiak J. Kawiak A. Schneider P. Kwasniewicz L. Polak N.. (2018a). New protocol for quantitative analysis of brain cortex electroencephalographic activity in patients with psychiatric disorders. Front. Neuroinformatics 12:27. 10.3389/fninf.2018.00027 29881339 PMC5976787

[B53] Wolpaw J. Wolpaw E. W. (2012). Brain-Computer Interfaces: Principles and Practice. New York, NY: Oxford University Press.

[B54] Zapała D. Zabielska-Mendyk E. Cudo A. Krzysztofiak A. Augustynowicz P. Francuz P. (2015). Short-term kinesthetic training for sensorimotor rhythms: effects in experts and amateurs. J. Mot. Behav. 47, 312–318. 10.1080/00222895.2014.982067 25514553

